# PIG3 promotes NSCLC cell mitotic progression and is associated with poor prognosis of NSCLC patients

**DOI:** 10.1186/s13046-017-0508-2

**Published:** 2017-03-04

**Authors:** Ming Li, Shanhu Li, Biao Liu, Meng-Meng Gu, Shitao Zou, Bei-Bei Xiao, Lan Yu, Wei-Qun Ding, Ping-Kun Zhou, Jundong Zhou, Zeng-Fu Shang

**Affiliations:** 1 School of Radiation Medicine and Protection, Medical College of Soochow University, Collaborative Innovation Center of Radiation Medicine of Jiangsu Higher Education Institutions, Suzhou, Jiangsu 215123 People’s Republic of China; 2 Laboratory of Medical Molecular Biology, Beijing Institute of Biotechnology, Beijing, 100850 People’s Republic of China; 3 Suzhou Cancer Center Core Laboratory, Nanjing Medical University Affiliated Suzhou Hospital, Suzhou, Jiangsu 215001 People’s Republic of China; 4 Department of Radiation Oncology, Simmons Comprehensive Cancer Center at UT Southwestern Medical Center, Dallas, 75390 TX USA; 5 Department of Pathology, University of Oklahoma Health Science Center, Oklahoma City, OK 73104 USA; 6 Department of Radiation Toxicology and Oncology, Beijing Institute of Radiation Medicine, Beijing, 100850 People’s Republic of China

**Keywords:** Non-small cell lung cancer (NSCLC), p53-induced gene 3 (PIG3), Mitotic progression, Microtubule assembly, Chemoresistance

## Abstract

**Background:**

Non-small cell lung cancer (NSCLC) is the most commonly diagnosed type of lung cancer that is associated with poor prognosis. In this study we explored the potential role of p53-induced gene 3 (PIG3) in the progression of NSCLC.

**Methods:**

Immunohistochemistry was used to determine the expression levels of PIG3 in 201 NSCLC patients. We performed in vitro studies and silenced endogenous PIG3 by using specific siRNAs that specific target PIG3. Immunofluorescent staining was performed to determine the effect of PIG3 on mitotic progression in NSCLC cells. The growth rates of microtubules were determined by microtubule nucleation analysis. Cell proliferation and chemosensitivity were analyzed by CCK8 assays. Annexin V staining and β-galactosidase activity analysis were used to evaluate PIG3 deficiency-related apoptosis and senescence, respectively.

**Results:**

PIG3 expression levels negatively correlated with overall survival and disease-free survival of NSCLC patients. Knock down of PIG3 resulted in repressed proliferation of NSCLC cells and increased aberrant mitosis, which included misaligning and lagging chromosomes, and bi- or multi-nucleated giant cells. In addition, PIG3 contributed to mitotic spindle assembly by promoting microtubule growth. Furthermore, loss of PIG3 sensitized NSCLC cells to docetaxel by enhancing docetaxel-induced apoptosis and senescence.

**Conclusions:**

Our results indicate that PIG3 promotes NSCLC progression and therefore suggest that PIG3 may be a potential prognostic biomarker and novel therapeutic target for the treatment of NSCLC.

**Electronic supplementary material:**

The online version of this article (doi:10.1186/s13046-017-0508-2) contains supplementary material, which is available to authorized users.

## Background

Worldwide, lung cancer is the deadliest type of cancer among both men and women [1, 2]. The most commonly diagnosed type of lung cancer is non-small cell lung cancer (NSCLC), which accounts for nearly 80% of lung cancer-related mortalities [1]. Despite recent advances in early diagnosis [3, 4], chemotherapy, and targeted biological molecular therapies [5, 6], the overall survival (OS) rate of patients with NSCLC is still significantly lower than that of many other solid tumors. Consequently, identifying novel biomarkers and elucidating the underlying mechanisms, which promote malignant progression of lung cancer, are desperately needed to improve lung cancer outcomes and provide personalized treatment.

The p53-induced gene 3 (*PIG3* or *TP53I3*) was initially identified through serial analysis of p53-target genes, which may be correlated with p53-mediated apoptosis [7]. The *PIG3* gene locates at chromosome 2p23.3 and comprises 5 exons [8]. The promoter of the *PIG3* gene includes a polymorphie pentanucleotide microsatellite sequence ((TGYCC)_n_, Y = C or T) that is the p53-binding cis-element and mediates p53-induced transactivation of *PIG3* [9]. (TGYCC)_15_ is the most common wild-type allele which localizes at *PIG3* promoter and has been reported to be correlated with a decreased risk of squamous cell carcinoma of the head and neck (SCCHN) [10]. Given that the PIG3 protein shares high sequence identity with NADH quinine oxidoreductase 1 (NQO1), it was implied that PIG3 may contribute to p53-induced cell apoptosis by promoting the production of reactive oxygen species (ROS) [7]. Consistent with this hypothesis, Porte and colleagues further investigated PIG3 3-D structure, substrate and cofactor specificity, and determined that PIG3 exhibits a NADPH-dependent reductase activity with orthoquinones [11]. PIG3 also acts as a ROS generator through direct association with and suppression of catalase in response to DNA damage [12]. The same group revealed that PIG3 is a novel regulator of DNA damage response [13]. Loss of PIG3 impairs recruitment of 53BP1, Mre11, Rad50, Nbs1 proteins to DNA break sites and attenuates DNA damage-induced phosphorylation of H2AX, Chk2 and Chk1 in response to UV treatment [13]. Our previous study found that PIG3 could enhance DNA-PKcs expression and contribute to Chk2, Chk1 phosphorylation after γ-ray exposure [14].

Given its established involvement in p53-induced apoptosis and DNA damage response, it seems reasonable to propose that PIG3 acts as a tumor suppressor to prevent cancer development and progression. In a recent study it was found that the tumor suppressor gene BRCA1 promotes transcription of PIG3 by p53 and that PIG3 expression status in breast cancer samples is positively correlated with OS rate of patients [15]. Research from other groups has demonstrated that PIG3 inhibits HIF-1α expression in renal cell carcinoma in addition to several other types of cancer cells in a mTOR pathway-dependent manner. Deficiency of PIG3 also promotes renal cancer cell migration by facilitating HIF-1α-VEGF signal pathway activation [16]. PIG3 is known to be highly expressed in papillary thyroid carcinoma (PTC) tissues and plays an oncogenic role by activating the PI3K/Akt pathway [17]. Although these seemingly contradictory reports indicated the potential importance of PIG3 in tumor progression, its role(s) in NSCLC still remains unknown and further investigation is warranted.

In the current study, we revealed that the expression levels of PIG3 in NSCLC tissues are inversely associated with OS and disease-free survival (DFS) of patients. To further explore the role of PIG3 in lung cancer development, we suppressed PIG3 expression in NSCLC cells and found that depletion of PIG3 leads to mitosis defects and an increase in the generation of bi- and multi-nucleus which might be due to the dysregulation of microtubule dynamic. Furthermore, we demonstrated that loss of PIG3 significantly increases NSCLC cells chemosensitivity to docetaxel, one of the most commonly used chemotherapeutic drugs against multiple cancers including advanced NSCLC [18], via enhancing docetaxel-induced apoptosis and senescence.

## Methods

### Patients and tissue specimens

Primary cancer tissue specimens obtained from 201 NSCLC patients were provided by the Nanjing Medical University Affiliated Suzhou Hospital (Suzhou, China). None of the patients underwent chemo- or radiotherapy prior to surgical resection. Clinicopathologic parameters and OS data were collected. Of all patients included in the study, 120 were male and 81 female. The average age of all patients was 59.7 years (range from 22 to 83 years) at the time of operation. Mean and median follow-up times after surgery were 47.3 and 38.0 months, respectively. The 5-year survival rate was 30.3%. Tumor tissue was routinely fixed in 10% phosphate-buffered formaldehyde and embedded in paraffin for immunohistochemical evaluation. This study was approved by the Ethics Committee of Nanjing Medical University Affiliated Suzhou Hospital. All patients signed informed consent.

### Immunohistochemistry and immunohistochemical evaluation

PIG3 localization was evaluated by immunohistochemistry (IHC) as described previously [19]. The PIG3 polyclonal antibody used was from Santa Cruz Biotechnology (Santa Cruz, CA, USA) and used at a 100-fold dilution.

PIG3 expression levels were scored blindly by two examiners who were unaware of the clinical characteristics. The staining area was scored as 0, 1, 2, 3 and 4 when 0–10, 11–25, 26–50, 51–75, and > 75% cells were stained positive, respectively. The staining intensity was scored as follows: 0, no staining; 1, pale yellow staining; 2, buffy staining; 3, intense brown staining. All scores were multiplied synchronically to calculate a subjective score for each section [20]. PIG3 expression levels were defined by a final score: low expression level (score ≤ 6) and high expression level (score > 6).

### Cell culture

RPMI-1640 medium supplemented with 10% fetal bovine serum was used to maintain NSCLC A549, H460 and H1299 cells. Cells were cultured in a humidified atmosphere at 37 °C and 5% CO_2_.

### PIG3 small interfering RNA and construct and transfection

PIG3-siRNA or non-targeting negative control (NC) siRNA were designed and synthesized by GenePharm (Shanghai, China). The sequences are listed in Table 1. The PIG3 constructs were generated by cloning PCR-amplified full-length human PIG3 cDNA into a pCMV-TAG-2B vector as described by Li B et al. [14]. Cells were seeded in 3.5 cm culture dishes when in logarithmic growth phase and were transiently transfected with 20 μM of PIG3-siRNA or NC siRNA or 5 μg PIG3 constructs or empty vector using Lipofectamine 3000 (Invitrogen, Carlsbad, CA, USA) following the manufacturer’s instructions. Following incubation at 37 °C for 48 h, cells were collected and lysed to verify the expression of PIG3 by Western blot analysis.

**Table 1:** SiRNA sequences for PIG3 and non-targeting negative control

siRNA name	Sequences	
	Sense (5′-3′)	Antisense (5′-3′)
siPIG3 #1	AAAUGUUCAGGCUGGAGACUATT	UAGUCUCCAGCCUGAACAUUUTT
siPIG3 #2	GGAAGUCUGAUCACCAGUUTT	AACUGGUGAUCAGACUUCCTT
siNC	UUCUCCGAACGUGUCACGUTT	ACGUGACACGUUCGGAGAATT

*NC* negative control

### Protein extraction and Western blot analysis

Protein extraction and Western blot were performed as previously described [19]. The following primary antibodies were used: PIG3 (Santa Cruz Biotechnology, Santa Cruz, CA, USA), PARP-1 (Cell Signaling Technology, Beverly, MA, USA) and GAPDH (Epitomics, Burlingame, CA, USA). All primary antibodies were used at a dilution of 1000-fold.

### CCK8 cell proliferation assay

Control and PIG3 knock down cells were cultured in a 96-well plate at a density of 1 × 10^4^ cells per well. Docetaxel (Taxotere; Sanofi-Aventis, Paris, France) was supplemented for increasing times (1, 2, 3, 4 and 5 days) or at indicated concentrations (2.5, 5, 10 and 20 μg/ml) for 48 h. After incubation with docetaxel, a volume of 10 μl Cell Counting Kit-8 solution (CCK8; Dojindo Laboratories, Japan) was added to each well and incubated for 2 h. The absorbance of each well was measured at 450 nm.

### Mitotic index analysis

PIG3 knock down and control cells were plated in 6 cm culture dishes, washed twice with PBS and fixed in 70% ethanol, diluted with PBS, for 24 h. Prior to staining, the cells were washed twice with PBS and permeabilized in 50 μl of 0.5% Triton X-100/PBS for 15 min. Cells were incubated with an anti-phosphorylated H3 (pSer10) antibody (1:100) (Cell Signaling Technology, Beverly, MA, USA) in PBS with 0.5% Triton X-100 at room temperature for 2 h and washed twice with PBS. Next, cells were incubated with an Alexa-488 conjugated anti-rabbit secondary antibody (Invitrogen, Carlsbad, CA, USA) at room temperature for 1 h. Cells were washed twice with PBS, treated with 1 μg/ml RNase A, stained with 25 μg/ml Propidium Iodide (PI) and analyzed by flow cytometry.

### Immunofluorescent staining and confocal microscopy

A549 cells and H460 cells were transfected with PIG3 or NC siRNAs. Cells were plated and cultured on poly-d-lysine-coated cover slides 48 h after transfection. Cells were washed twice in PBS and fixed in 4% paraformaldehyde/PBS at room temperature for 30 min. Next, the cells were permeabilized with 0.5% Triton X-100/PBS at room temperature for 15 min. After permeabilization, cells were blocked with 1% bovine serum albumin/PBS at room temperature for 30 min. Immunostaining was performed by incubating with anti-α-tubulin antibody, γ-tubulin (Sigma, St Louis, MO, USA) and phosphorylated H3 (pSer10) (Cell Signaling Technology, Beverly, MA, USA) antibodies (1:1000) for 4 h at room temperature. After incubating with the primary antibodies, cells were washed three times with PBS. Cells were then incubated with Alexa-488 conjugated anti-rabbit and Alexa-568 conjugated anti-mouse secondary antibodies (Invitrogen, Carlsbad, CA, USA) for 1 h at 37 °C. For visualization of DNA, 4, 6-diamidino-2-phenylindole (DAPI, Vector Laboratories, Burlingame, CA, USA) was added to the mounting medium. Images were obtained using a LSM 510 laser-scanning confocal microscope (Zeiss, Germany).

### Microtubule regrowth assay

Microtubule regrowth assays were performed as previously described [21]. PIG3 depletion and control A549 cells were cultured on cover slides coated as before and incubated with ice-cold medium supplemented with 1 μg/ml nocodazole (Sigma, St Louis, MO, USA) for 1 h. Fresh medium without nocodazole was added after washing with PBS. Cells were fixed in 4% paraformaldehyde/PBS and subjected to immunostaining as described above.

### Apoptosis detection

For the detection of apoptosis, the FITC Annexin V Apoptosis Detection Kit (BD, Pharmingen, San Diego, CA, USA) was used according to the manufacturer’s protocol. PIG3 silenced or control cells were treated with or without docetaxel as described above and harvested. The cells were washed thrice with PBS and resuspended in 1× binding buffer at a concentration of 1 × 10^6^ cells/ml. The cell suspension (100 μl) was transferred to a new tube and 5 μl of FITC-conjugated Annexin V was added. Cells were incubated for 15 min at room temperature. After addition of 400 μl of 1× binding buffer, cells were analyzed by flow cytometry.

### Senescent cells detection

To detect senescent cells, the senescence-associated β-galactosidase (SA-β-Gal) assay was performed. PIG3 silenced or control cells were cultured in 6-well plates at a density of 20,000 cells/well and treated with or without docetaxel at indicated concentrations and times. Cells were fixed and stained following the Senescence β-gal Staining Kit manufacturer’s protocol (Cell Signaling Technology, Beverley, MA, USA). Cells were incubated for 16 h at 37 °C in a dry incubator without CO_2_ after which blue stained cells were detected under a bright field microscope (Leica Corporation, Germany).

### Statistical analysis

Statistical analyses were conducted using SPSS 19.0 software (SPSS Inc., Chicago, IL, USA). For continuous or discrete data analysis, the chi-square test was used. The association between PIG3 expression and OS or DFS rates was estimated by Kaplan-Meier survival analysis and assessed using a log-rank test. The effect of clinicopathologic variables on survival was assessed with a Cox regression model. One-way ANOVA was performed for multiple comparisons. The data were presented as the mean ± standard deviation (SD) of three independent experiments. All tests were 2-sided, and differences were considered significant when *P* < 0.05.

## Results

### Increased expression of PIG3 is associated with poor prognosis of NSCLC patients

**Fig. 1:**
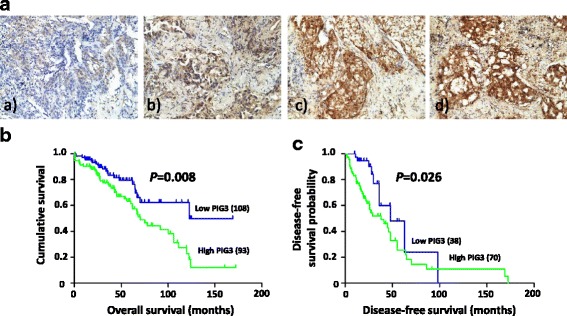
PIG3 expression is associated with poor prognosis of NSCLC patients. **a** Immunohistochemical analysis of PIG3 in 201 tumor tissue samples (100×): *a*), Negative expression; *b*), Low expression; *c*), Moderate expression; *d*), High expression. **b** Kaplan-Meier survival analysis between PIG3 expression levels and overall survival of NSCLC patients (*P* = 0.008). **c** The association between PIG3 expression levels and disease-free survival of NSCLC patients (*P* = 0.026). All groups were ranked according to PIG3 staining intensity

To investigate the potential role of PIG3 in the progression of NSCLC, we performed PIG3 immunohistochemistry (IHC) in NSCLC tissue obtained from 201 patients (Fig. 1a). The clinicopathologic characteristics of all patients are listed in Table 2. Our results showed that an upregulated PIG3 expression level significantly correlated with tumor size (*P* = 0.0003), differentiation degree (*P* = 0.004), pathological stage (*P* = 0.004) and distant metastasis (*P* = 0.001). In addition, no association could be observed between PIG3 expression and age, gender or relapse.

**Table 2:** Correlation between PIG3 expression and clinicopathological parameters of 201 patients with non-small cell lung cancer (NSCLC)

Characteristics	Total (*n* = 201)	PIG3 protein expression		*P*-value
		Low	High	
Age				
≤60	95	53	42	>0.05
>60	106	55	51	
Gender				
Male	120	60	60	>0.05
Female	81	48	33	
Smoking history				
Yes	136	80	56	<0.05
No	65	28	37	
Tumor size				
<3 cm	105	69	36	<0.01
≥3 cm	96	39	57	
Differentiation degree				
Poorly	78	32	46	<0.01
Well, moderately	123	76	47	
Pathological stage				
IA	143	88	55	<0.01
IB	58	20	38	
Distant metastasis				
Yes	60	22	38	<0.01
No	141	86	55	
Relapse				
Yes	20	12	8	>0.05
No	181	96	85	

To evaluate the relationship between PIG3 expression and patient prognosis, Kaplan-Meier survival analysis for OS and DFS were performed (Fig. 1b, c). Our results indicated that patients with a higher PIG3 expression (high PIG3) demonstrate a significantly shorter OS (*P* = 0.008) and DFS (*P* = 0.026) compared to patients with low PIG3 expression (low PIG3). Moreover, multivariate Cox regression analysis showed high PIG3 to be an independent prognostic marker for low survival that was associated with a relative risk of 1.742 (Table 3; 95% CI 1.023–2.976; *P* = 0.041). Together, these findings suggested that PIG3 may have an oncogenic role in the progression of lung cancer.

**Table 3:** Multivariate analysis of cancer specific survival

	HR	95% CI	*P*-value
Gender	0.901	0.479–1.694	0.746
Age	1.410	0.849–2.342	0.185
Smoking	0.685	0.353–1.330	0.264
Recurrence	1.417	0.705–2.847	0.328
Metastasis	2.375	1.428–3.952	0.001
PIG3 expression (high *vs*. low)	1.742	1.023–2.967	0.041

*HR* hazard ratio, *CI* confidence interval

### Suppression of PIG3 results in bi- and multi-nucleated cells and retarded growth of NSCLC cells

**Fig. 2:**
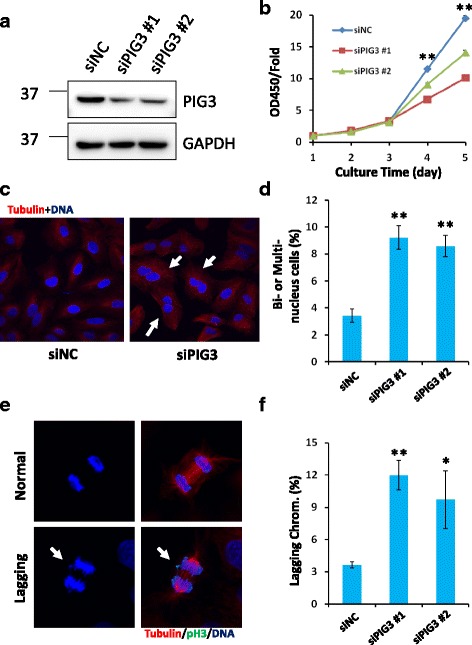
Loss of PIG3 leads to increased outcome of bi-/multi-nucleated cells and aberrant chromosomes segregation in normal cultured cells. **a** Western blot analysis demonstrating the efficacy of two different siRNAs against PIG3 in A549 cells at 48 h post-transfection. **b** 3× 10^3^ cells were seeded in 96-well plates at day 0, and CCK8 assay was used to determine cell proliferation rates at indicated days (1, 2, 3, 4 and 5 days). Absorbance values at 450 nm were normalized by the value measured on day 1 (^**^ *P* < 0.01). **c** Representative images showing bi- and multinucleated cells (*arrowheads*). **d** Quantitative analysis of bi- and multi-nucleated cells from PIG3-deficient and control A549 cells (^**^ *P* < 0.01). **e** Representative images showing normal and aberrant anaphase cells with lagging chromosome (*arrowheads*). **f** Percentages of mitotic cells showing lagging chromosome were counted from three independent experiments, ^**^ *P* < 0.01, ^*^ *P* < 0.05

To determine the role of PIG3 on the progression of NSCLC, two different siRNA constructs that target PIG3 and a NC siRNA were transfected into A549 NSCLC cells. Western blot analysis verified that both siRNAs significantly suppress endogenous PIG3 protein expression in A549 cells (Fig. 2a). We used the siRNA with the highest efficacy to transfect H460 cells and found that PIG3 expression was significantly silenced in H460 cells (Additional file 1: Figure S1a). Compared with corresponding NC groups, depletion of PIG3 significantly reduced the proliferation rates of A549 and H460 cells (Fig. 2b and Additional file 1: Figure S1b). The *PIG3* is one of p53 target genes. Consistent with this, PIG3 expression is suppressed in H1299 cells which have the homozygous partial deletion of the TP53 gene. We observed that overexpression of PIG3 dramatically promotes growth speed of H1299 cells (Additional file 2: Figure S2a and b). Interestingly, we found an increase in the generation of giant cells in PIG3 deficient NSCLC cells. After staining with an anti-α-tubulin antibody and DAPI to visualize DNA, numerous giant cells with bi- or multi-nuclei were observed in PIG3 deficient-A549 cells (Fig. 2c, d). Bi- and multi-nucleated cells may arise from cytokinesis failure, which is commonly induced by abnormality of chromosomal segregation [22, 23]. Consistent with this observation, we identified a significant increase of chromosomes lagging during anaphase in PIG3 deficient NSCLC cells as compared to NC cells (Fig. 2e, f). In a recent study, it was shown that failure in cell cleavage induces cellular senescence [24]. Here, we found that giant cells induced by PIG3 deficiency exhibited activated SA-β-galactosidase (Fig. 5e, f), which is indicative of a senescent phenotype.

### PIG3 contributes to mitotic spindle assembly in NSCLC cells

**Fig. 3:**
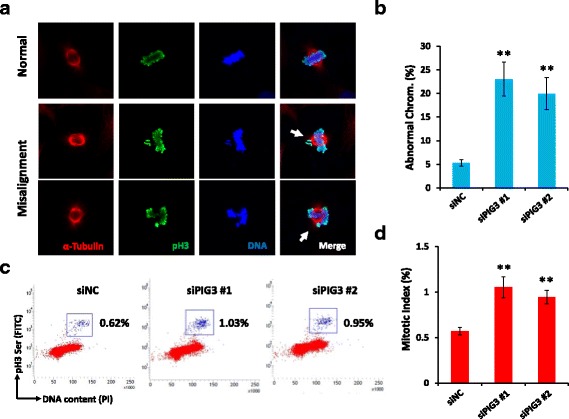
PIG3 is required for normal mitotic progression. **a** Exponentially growing PIG3 depleted and control A549 cells were subjected to immunofluorescent analysis using the indicated antibody. The representative images show aberrant mitotic cells with misalignment chromosomes (*arrowheads*). **b** Percentages of mitotic cells showing misaligned chromosomes were counted from three independent experiments (^**^ *P* < 0.01). **c** A549 cells were transfected with control siRNA or PIG3 siRNAs for 48 h and stained with an anti-phosphorylated H3 antibody to determine the percentage of mitosis cell population by flow cytometry. **d** Quantitative analysis of mitotic index. Results were generated from three independent experiments (^**^ *P* < 0.01)

The observation of increased abnormality of chromosome segregation in PIG3 deficient cells strongly indicated that PIG3 may play an important role in the regulation of spindle organization. During the transition from prometaphase to metaphase, kinetochores are held by microtubules that are released from the opposite sites of centrosomes and allow chromosomes to align along the metaphase plate of the spindle apparatus. To define whether or not PIG3 plays a role in mitotic spindle organization, NSCLC cells were stained with antibodies directed against α-tubulin and a mitosis marker, phosphorylated histone 3, at the Ser10 site. We found that, in both A549 and H460 cells, PIG3 depletion resulted in a noticeable increase of chromosome misalignment (Fig. 3a, b and Additional file 1: Figure S1c, d). Consistent with this observation, we found an increase in mitotic index in NSCLC cells lacking PIG3 as compared to the control cells (Fig. 3c, d). Thus, aberrant spindle assembly and mitotic progression may potentially lead to mitotic arrest.

### Lack of PIG3 inhibits microtubule dynamics in NSCLC cells

**Fig. 4:**
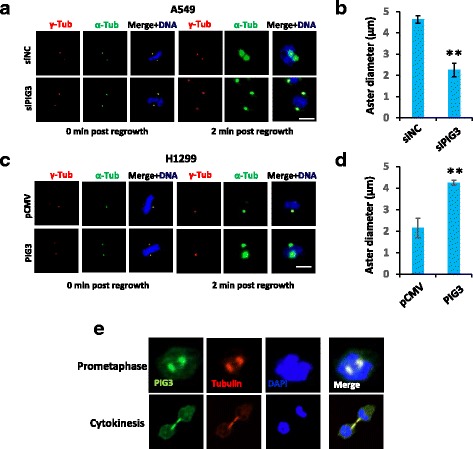
PIG3 is required for microtubule growth in NSCLC cells. **a** Knock down of PIG3 by siRNA markedly inhibits the regrowth of microtubules from centrosomes in A549 cells. PIG3 and control siRNAs were transfected into A549 cells and 48 h post transfection the cells were treated with chilled medium + 1 μM nocodazole for an additional 1 h on the ice. Cells were fixed and stained with an anti-α-tubulin antibody. **b** The length of the microtubule emanating from the centrosomes was measured (*n* ≥ 50, ^**^ *P* <0.01). **c** Overexpressing PIG3 significantly increases microtubules growth rates in H1299 cells. **d** The length of the microtubule emanating from the centrosomes was measured (*n* ≥ 50, ^**^ *P* <0.01). **e** The localization of PIG3 in the cells detected by immunofluorescent staining

Abnormal microtubule dynamics regulation has been reported to be correlated with aberrant mitotic organization [25]. To identify whether or not PIG3 is involved in microtubule organization, PIG3 silenced and control A549 cells, PIG3 overxpressed and empty vector control H1299 cells were incubated with ice-cold medium including high levels of nocodazole to depolymerize microtubules. Microtubules started to regrow after removal of the nocodazole-containing medium. Microtubule nucleation speed was analyzed by the diameter of the microtubule asters that were grown from centrosomes (Fig. 4a, c). We observed that, in A549 cells, loss of PIG3 significantly inhibited microtubule nucleation (Fig. 4b), whereas increased PIG3 in H1299 cells promotes microtubule regrowth rate (Fig. 4d). Consistent with its role in microtubule dynamic regulation, we determined that PIG3 co-localized with tubulin and accumulated at the spindle apparatus during mitosis (Fig. 4e). PIG3 localizes at both nucleus and cytoplasm in interphase cells and can be recruited at DNA damage sites when cells were exposed to γ ray (Additional file 3: Figure S3), which is consistent with the previous report [13]. In conclusion, our data revealed for the first time a novel role of PIG3 in microtubule regulation.

### Depletion of PIG3 sensitizes NSCLC cells to docetaxel

**Fig. 5:**
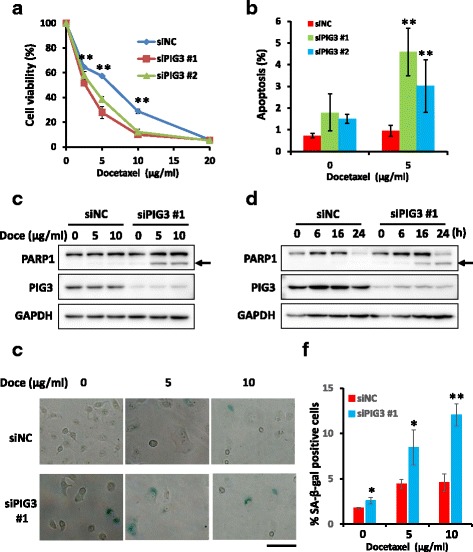
Depletion of PIG3 sensitizes NSCLC cells to docetaxel through promoting apoptosis and senescence. **a** Forty-eight hrs following transfection with PIG3 and control siRNAs, A549 cells were exposed to various concentrations of docetaxel. Cell proliferation was determined by CCK8 assay 48 h post treatment. The data are expressed as the mean and standard deviations from three independent experiments (^**^ *P* < 0.01). **b** Forty-eight hrs following transfection with PIG3 and control siRNAs, A549 cells were treated with 5 μg/ml docetaxel or DMSO for 24 h. Apoptotic cells were detected using the Annexin V staining method. The data are expressed as mean and standard deviations from three independent experiments, ^**^ *P* < 0.01 as compared to control siRNA transfected cells under similar treatment conditions of docetaxel. **c** and **d** Forty-eight hrs following transfection with PIG3 and control siRNAs, A549 cells were treated with different concentrations (0, 5 and 10 μg/ml) of docetaxel for 24 h or 5 μg/ml of docetaxel for indicated time intervals (0, 6, 16 and 24 h). Apoptosis was determined by PARP-1 cleavage (indicated by an *arrow*) following Western blot analysis. **e** SA-β-gal staining of NSCLC cells. PIG3 depleted and control A549 cells were treated with 0, 5 and 10 μg/ml of docetaxel for 48 h and then stained for SA-β-gal activity. **f** Quantitative analysis of senescent cells. The results were generated from three independent experiments (^*^ *P* < 0.05, ^**^ *P* < 0.01)

Our data showed that PIG3-depleted cells exhibit aberrant mitosis, which may be associated with dysregulation of the dynamics of microtubules, similar to that of drug-treated microtubule cells [26]. Next, we investigated whether silencing of PIG3 expression may modulate the sensitivity of NSCLC cells to docetaxel, a well-established anti-mitotic chemotherapeutic drug that is used to treat advanced NSCLC [18]. The proliferation of PIG3 knock down and control NSCLC cells was accessed by CCK8 assay at 48 h after docetaxel treatment (2.5, 5, 10 and 20 μg/ml). Our data showed that PIG3-depleted cells were more sensitive to docetaxel treatment compared to NC cells, and PIG3-overexpression increased NSCLCs resistance to docetaxel (Fig. 5a, Additional file 2: Figure S2c and Additional file 4: Figure S4). Docetaxel-induced apoptosis was determined by flow cytometry. As shown in Fig. 5b, treatment with 5 μg/ml docetaxel dramatically induces apoptosis in PIG3-silenced but not in control A549 cells. Docetaxel-induced apoptosis was verified by cleaved PARP-1 immunoblot analysis. We found that docetaxel treatment caused severe PARP-1 cleavage and apoptosis in PIG3-depleted cells but not in control cells (Fig. 5c, d). In addition, PIG3 knock down and control A549 cells were subjected to SA-β-gal staining. Our results indicated that following docetaxel treatment PIG3 depletion significantly enhanced senescence (Fig. 5e, f), whereas PIG3 overexpression prevents docetaxel induced senescence in H1299 cells (Additional file 2: Figure S2d).

## Discussion

Due to its important role in ROS generation and p53-mediated apoptosis, PIG3 is well-known as an inhibitory factor of cancer cell survival [27]. It has been reported that PIG3 promotes proliferation of PTC cells by activating the PI3K/Akt signaling pathway [17]. Here, we examined the expression of PIG3 in 201 NSCLC samples and found that expression levels of PIG3 positively associated with poor prognosis of NSCLC patients (Fig. 1b, c). Consistent with these results, knocking down PIG3 significantly inhibited proliferation of NSCLC cells (Fig. 2b, Additional file 1: Figure S1b). Furthermore, we revealed an important role of PIG3 in spindle stability maintenance and mitotic progression regulation. It is well known that numerous mitotic regulators are overexpressed in various tumors due to the increased proliferation of cancer cells compared to healthy cells [28–30]. Overall, our study provides evidence that PIG3 may contribute to NSCLC development by promoting mitotic progression.

We have previously reported the positive correlation of DNA-PKcs protein levels with PIG3 expression [14]. DNA-PKcs is a critical kinase that is involved in the non-homologous end joining (NHEJ) pathway and repairs DNA double strand breaks (DSBs). We and others have identified a novel role of DNA-PKcs in maintaining normal spindle formation or centrosomes stability [21, 31–33]. In addition, DNA-PKcs contributes to mitotic entry and cytokinesis progression by activating Plk1 in a temporal and spatial fashion [34, 35]. During mitosis, the Chk2-Brca1 signaling cascade is downstream of DNA-PKcs and has an effect on mitotic microtubule assembly [21]. Consistent with these important roles, inactivation of DNA-PKcs results in a phenotype of abnormal mitotic spindle organization and mitotic catastrophe, which is accompanied by dysregulation of Plk1 and Chk2 in mitosis [21, 34, 36]. Similar to the loss of DNA-PKcs, we found that there is an increase in misaligned and lagged chromosomes in PIG3 deficient cells compared to normal cells, which implies a functional link between these two molecules (Figs. 2 and 3). In conclusion, further investigation is warranted to delineate the impact of PIG3 on the mitotic function of Chk2, Plk1 and whether expression of DNA-PKcs will restore the defects found during mitosis that were induced by PIG3 deficiency.

Appropriate microtubule assembly is crucial for mitotic spindle organization, proper chromosome alignment and their segregation. In a recent study, Ertych et al. demonstrated that increased assembly rates by Aurora A-overexpressing or loss of Chk2 in colon cancer cells resulted in abnormal microtubule spindle geometry and a subsequent increase of chromosomal instability (CIN), which is one of the characteristics of tumor cells and a driving force for cancer development [37, 38]. In addition, CIN plays a critical role in lung cancer progression and fluorescence in situ hybridization (FISH) analysis revealed a close association between CIN in NSCLC and poor prognosis of patients [39, 40]. Consistent with these findings, our results indicate that PIG3 positively regulated microtubule growth rate (Fig. 4). These studies support the speculation that increased PIG3 expression promoted aggressive progression of NSCLC by accelerating microtubule assembly and the generation of CIN cells. As described previously, PIG3 is homologous with NADH quinine oxidoreductase 1 (NQO1) [7]. In human cells, NQO1 has been reported to localize to the mitotic spindle [41]. Furthermore, NQO1 has a direct physical interaction with the mitotic factor Aurora A and promotes Aurora A degradation by antagonizing the protective function of TPX2 [42]. Further investigation of the potential interaction between PIG3 and the TPX2-Aurora A signal pathway during mitosis as well as its potential role(s) in regulating microtubule assembly and chromosomal stability is clearly warranted.

Microtubule dynamics-targeted chemotherapeutic agents such as taxanes are widely used, alone or in combination with other drugs, to treat various solid tumors such as advanced NSCLC [43–45]. Unfortunately, primary resistance to these agents is a major challenge for the use of taxanes in clinical applications. Numerous studies have shown that targeting the mitotic factors may be a successful strategy to overcome the chemoresistance to taxanes in cancer cells [30, 45]. In the present study, we found that a lack of PIG3 significantly sensitizes NSCLC cells to docetaxel, which at least can partially be attributed to the increased induction of apoptosis and senescence. This suggested that docetaxel may be considered an effective targeted therapy against PIG3-low expressing lung cancers. In this context, it is important to further examine whether low PIG3 expression is associated with clinical benefits patients with NSCLC following docetaxel-based chemotherapy.

## Conclusion

In summary, in our work we revealed that PIG3 expression levels positively correlated with poor prognosis of NSCLC patients. Our study also demonstrated that PIG3 is a novel mitotic factor that regulated microtubule dynamics and mitotic spindle assembly. In addition, loss of PIG3 induced phenotypes of mitotic catastrophe, which exhibited misaligned and lagged chromosomes and led to multi-nucleated and senescent cells. Loss of PIG3 conferred sensitivity of NSCLC cells to docetaxel-based chemotherapy. These findings demonstrated a role for PIG3 in the progression of NSCLC, indicating that PIG3 may be a potential prognostic marker and novel therapeutic target for NSCLC.

## Additional files

Additional file 1: Figure S1. Loss of PIG3 inhibites cell proliferation and leads to increased outcome of misalignment chromosomes in H460 NSCLC cells. **a** Western blot analysis demonstrating the efficacy of PIG3 siRNA #1 in H460 cells at 48 h post-transfection. **b** 3× 10^3^ cells were seeded in 96-well plates at day 0, and CCK8 assay was used to determine cell proliferation rates at indicated days (1, 2, 3, 4 and5 days). Absorbance values at 450 nm were normalized by the value measured on day 1 (^*^ *P* < 0.05, ^**^ *P* < 0.01). **c** Exponentially growing PIG3 depleted and control H460 cells were subjected to the immunofluorescent staining using the indicated antibody. The representative images showing aberrant mitotic cells with misalignment chromosomes (arrowheads). **d** Percentages of mitotic cells showed misaligned chromosomes were counted from three independent experiments. ^**^ *P* < 0.01. (PPT 3533 kb)
Additional file 2: Figure S2. Overexpression PIG3 promotes NSCLC cells proliferation and increases resistance of NSCLC cells to docetaxel. **a** Western blot analysis demonstrating the level of PIG3 in PIG3-overexpressed and control H1299 cells. **b** 3× 10^3^ cells were seeded in 96-well plates at day 0, and CCK8 assay was used to determine cell proliferation rates at indicated days (1, 2, 3, 4 and 5 days). Absorbance values at 450 nm were normalized by the value measured on day 1 (^*^ *P* < 0.05, ^**^ *P* < 0.01). **c** PIG3 overexpressed and control H1299 cells were exposed to various concentrations of docetaxel. Cell proliferation was determined by CCK8 assay 48 h post treatment. The data are expressed as the mean and standard deviations from three independent experiments (^**^ *P* < 0.01). **d** PIG3 overexpressed and control H1299 cells were treated with 0, 5 and 10 μg/ml of docetaxel for 48 h and then stained for SA-β-gal activity. Quantitative analysis of senescent cells. The results were generated from three independent experiments (^*^ *P* < 0.05). (PPT 298 kb)
Additional file 3: Figure S3. The localization of PIG3 in the cells detected by immunofluorescent staining. A549 cells were treated or untreated with 4Gy γ ray irradiation. One hour post irradiation, cells were fixed and stained using anti-PIG3, KAP-1 and phosphorylated H2AX antibody. (PPTX 1444 kb)
Additional file 4: Figure S4. Depletion of PIG3 sensitized NSCLC cells to docetaxel. Forty eight hrs following transfection with PIG3 and control siRNAs, H460 cells were exposed to various concentrations of docetaxel. Cell proliferation was determined by CCK8 assay 48 h post treatment. The data are expressed as the mean and standard deviations from three independent experiments (^**^ *P* < 0.01). (PPT 475 kb)
