# Supplementary material for: PIG3 promotes NSCLC cell mitotic progression and is associated with poor prognosis of NSCLC patients
Source: J Exp Clin Cancer Res. 2017 Mar 4;36:39. doi: 10.1186/s13046-017-0508-2 (PMC5336678; doi:10.1186/s13046-017-0508-2)
Supplement: Additional file 1: Figure S1. — Loss of PIG3 inhibites cell proliferation and leads to increased outcome of misalignment chromosomes in H460 NSCLC cells. a Western blot analysis demonstrating the efficacy of PIG3 siRNA #1 in H460 cells at 48 h post-transfection. b 3× 10^3^ cells were seeded in 96-well plates at day 0, and CCK8 assay was used to determine cell proliferation rates at indicated days (1, 2, 3, 4 and5 days). Absorbance values at 450 nm were normalized by the value measured on day 1 (* P < 0.05, ** P < 0.01). c Exponentially growing PIG3 depleted and control H460 cells were subjected to the immunofluorescent staining using the indicated antibody. The representative images showing aberrant mitotic cells with misalignment chromosomes (arrowheads). d Percentages of mitotic cells showed misaligned chromosomes were counted from three independent experiments. ** P < 0.01. (PPT 3533 kb) [file 13046_2017_508_MOESM1_ESM.ppt]

## Slide 1
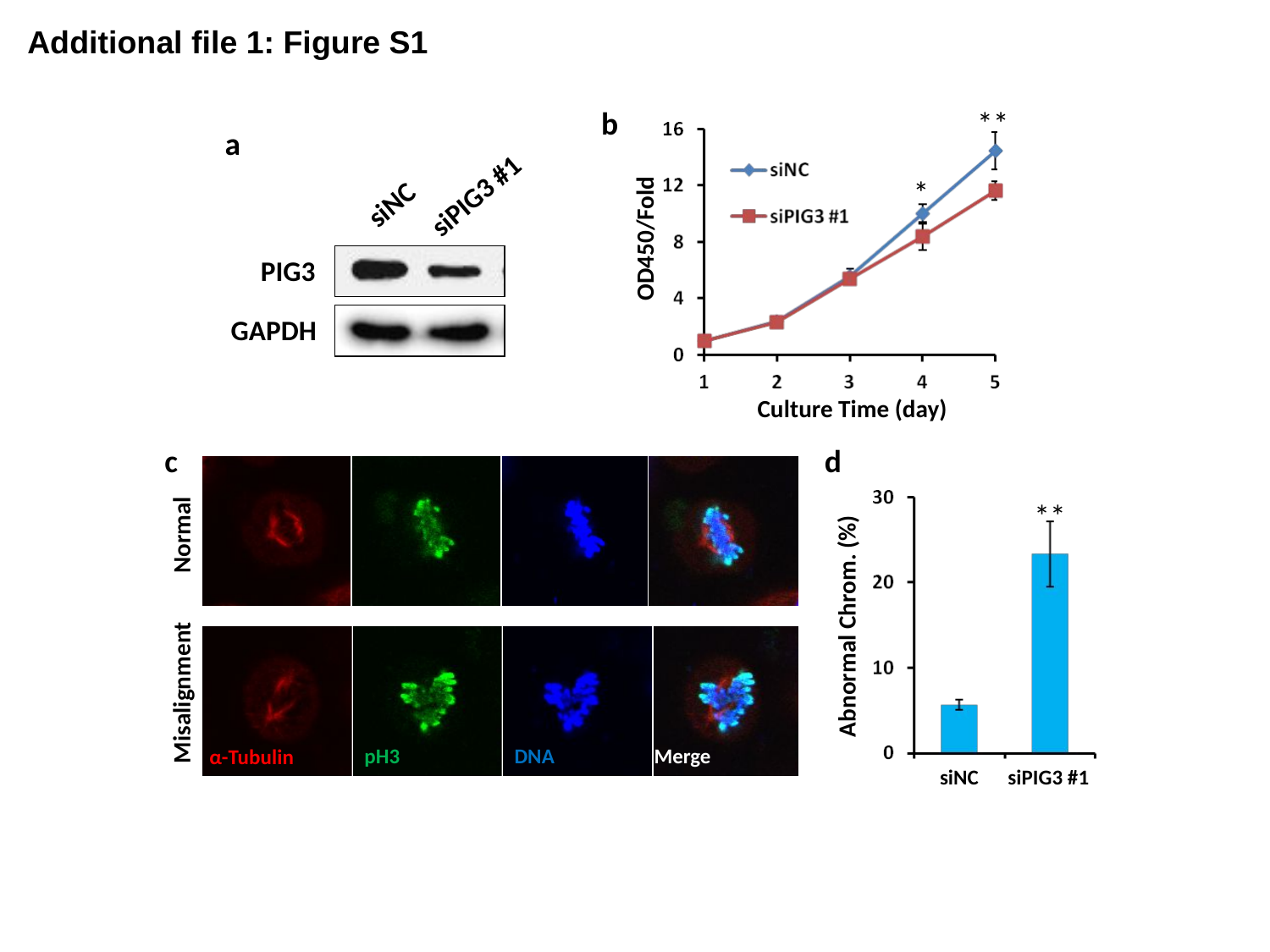

Additional file 1: Figure S1
b
**
a
*
OD450/Fold
siPIG3 #1
siNC
PIG3
GAPDH
Culture Time (day)
c
d
**
Normal
 Abnormal Chrom. (%)
Misalignment
pH3
DNA
Merge
α-Tubulin
siNC
siPIG3 #1
