# Supplementary material for: PIG3 promotes NSCLC cell mitotic progression and is associated with poor prognosis of NSCLC patients
Source: J Exp Clin Cancer Res. 2017 Mar 4;36:39. doi: 10.1186/s13046-017-0508-2 (PMC5336678; doi:10.1186/s13046-017-0508-2)
Supplement: Additional file 2: Figure S2. — Overexpression PIG3 promotes NSCLC cells proliferation and increases resistance of NSCLC cells to docetaxel. a Western blot analysis demonstrating the level of PIG3 in PIG3-overexpressed and control H1299 cells. b 3× 10^3^ cells were seeded in 96-well plates at day 0, and CCK8 assay was used to determine cell proliferation rates at indicated days (1, 2, 3, 4 and 5 days). Absorbance values at 450 nm were normalized by the value measured on day 1 (* P < 0.05, ** P < 0.01). c PIG3 overexpressed and control H1299 cells were exposed to various concentrations of docetaxel. Cell proliferation was determined by CCK8 assay 48 h post treatment. The data are expressed as the mean and standard deviations from three independent experiments (** P < 0.01). d PIG3 overexpressed and control H1299 cells were treated with 0, 5 and 10 μg/ml of docetaxel for 48 h and then stained for SA-β-gal activity. Quantitative analysis of senescent cells. The results were generated from three independent experiments (* P < 0.05). (PPT 298 kb) [file 13046_2017_508_MOESM2_ESM.ppt]

## Slide 1
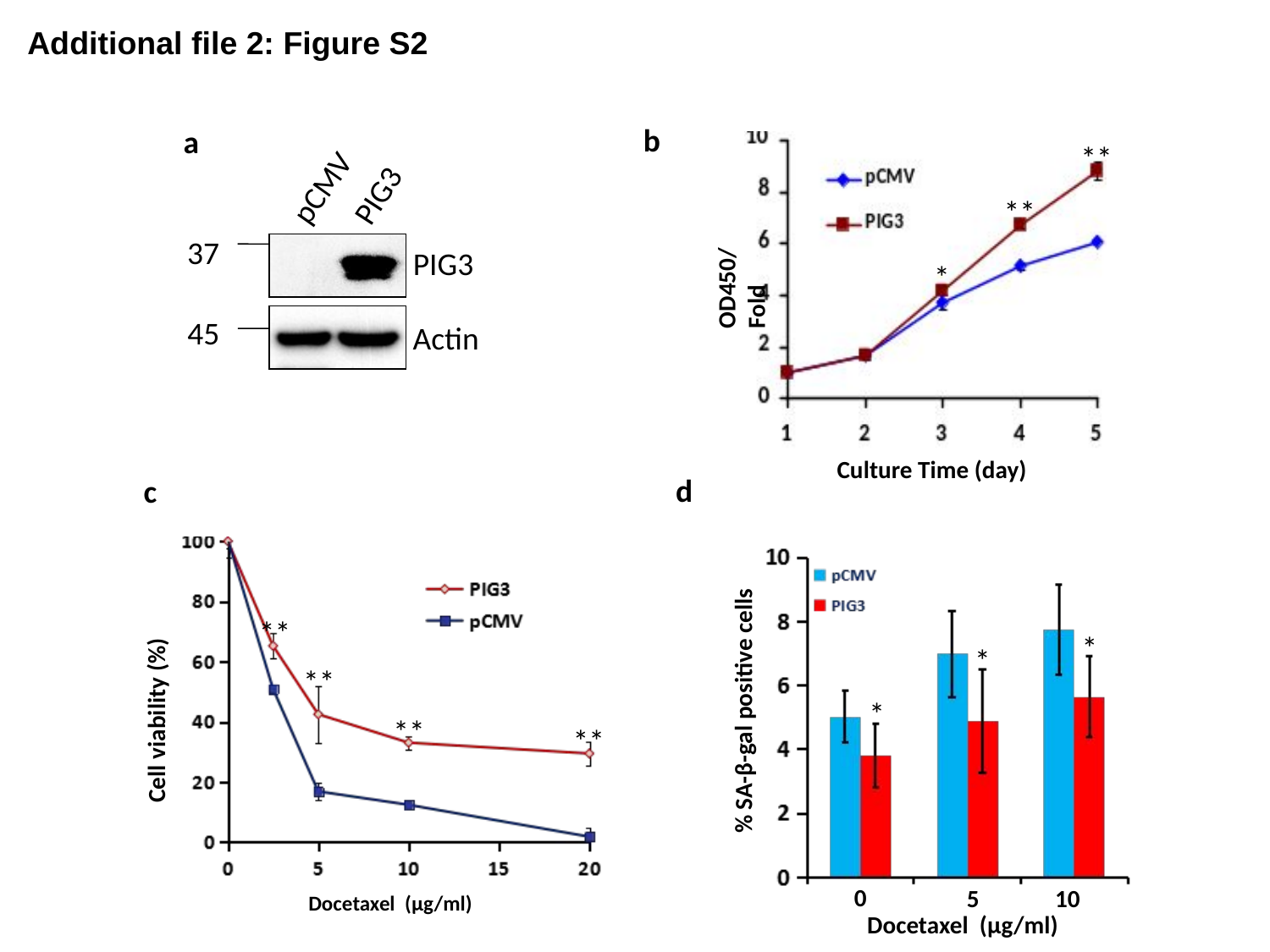

Additional file 2: Figure S2
b
a
pCMV
PIG3
37
PIG3
45
Actin
**
**
OD450/Fold
*
Culture Time (day)
d
c
**
*
*
**
*
% SA-β-gal positive cells
Cell viability (%)
**
**
0
5
10
Docetaxel (μg/ml)
Docetaxel (μg/ml)
