# Supplementary figures and images for: PIG3 promotes NSCLC cell mitotic progression and is associated with poor prognosis of NSCLC patients
Source: J Exp Clin Cancer Res. 2017 Mar 4;36:39. doi: 10.1186/s13046-017-0508-2 (PMC5336678; doi:10.1186/s13046-017-0508-2)

## Slide 1
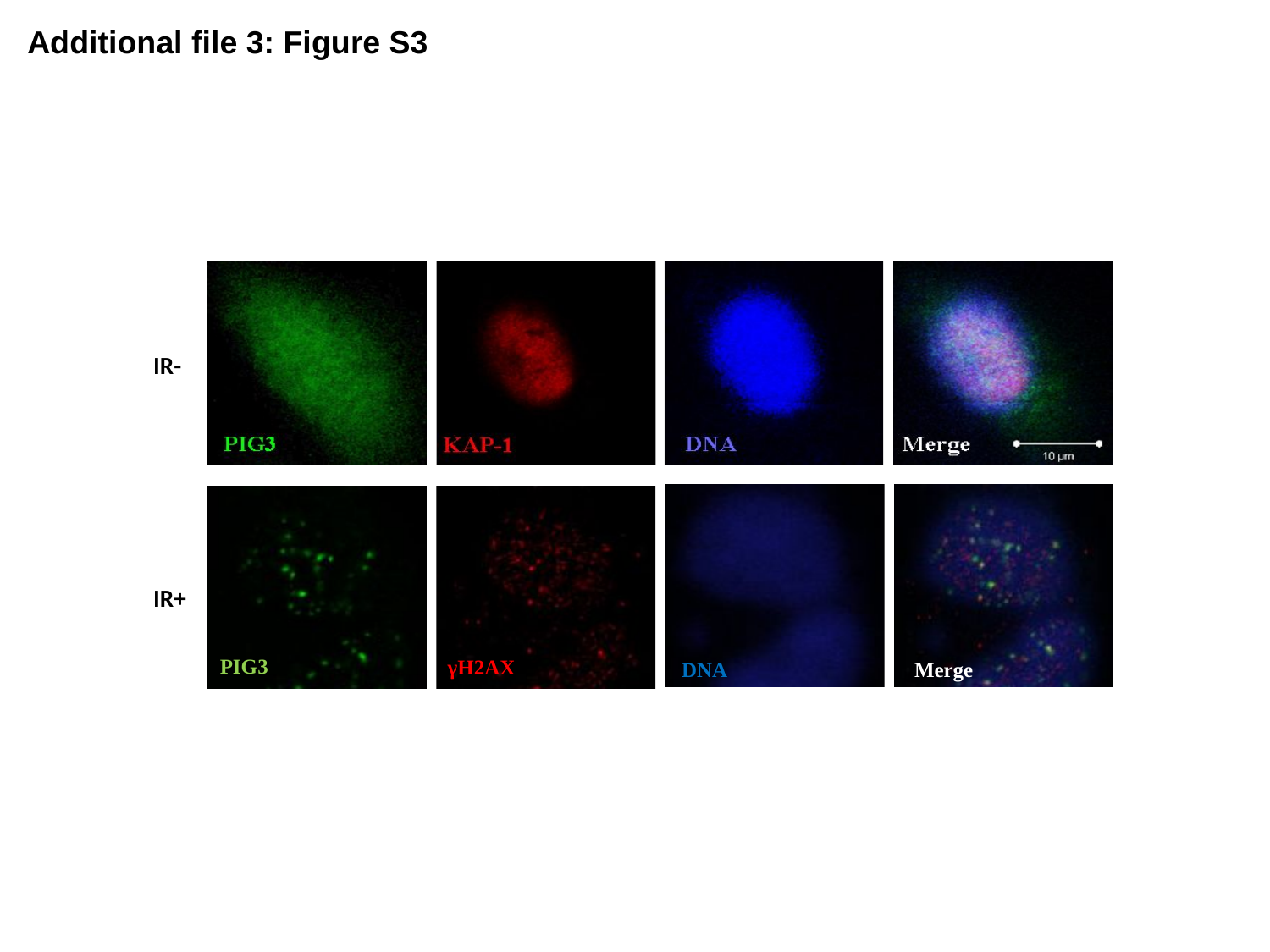

Additional file 3: Figure S3
IR-
IR+
PIG3
γH2AX
DNA
Merge

Supplement: Additional file 3: Figure S3. — The localization of PIG3 in the cells detected by immunofluorescent staining. A549 cells were treated or untreated with 4Gy γ ray irradiation. One hour post irradiation, cells were fixed and stained using anti-PIG3, KAP-1 and phosphorylated H2AX antibody. (PPTX 1444 kb) [file 13046_2017_508_MOESM3_ESM.pptx]

## Slide 1
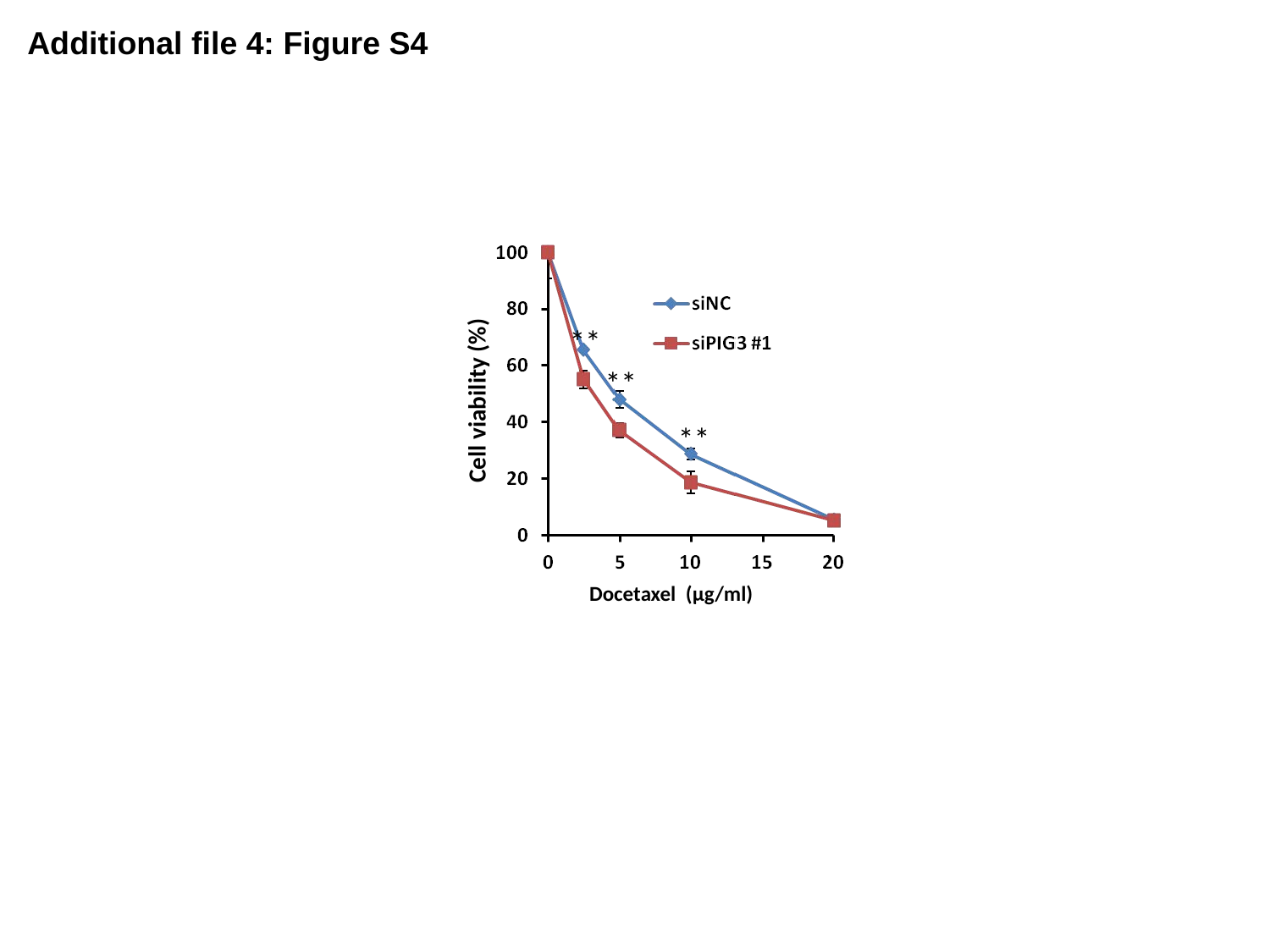

Additional file 4: Figure S4
**
**
Cell viability (%)
**
Docetaxel (μg/ml)

Supplement: Additional file 4: Figure S4. — Depletion of PIG3 sensitized NSCLC cells to docetaxel. Forty eight hrs following transfection with PIG3 and control siRNAs, H460 cells were exposed to various concentrations of docetaxel. Cell proliferation was determined by CCK8 assay 48 h post treatment. The data are expressed as the mean and standard deviations from three independent experiments (** P < 0.01). (PPT 475 kb) [file 13046_2017_508_MOESM4_ESM.ppt]
